# Management for Active Infective Endocarditis with extensive aortic root abscess

**DOI:** 10.1186/1749-8090-10-S1-A212

**Published:** 2015-12-16

**Authors:** Takeo Tedoriya, Ryoi Okano, Kenichi Kamiya, Satoru Maeba, Masaomi Fukuzumi

**Affiliations:** 1 Division of Cardiovascular Surgery, Cardiovascular Center, Ageo Central General Hospital, Ageo, 3628588, Japan

## Background/Introduction

Active Aortic Valve Infective Endocarditis with root abscess has had high mortality and morbidity, especially the infective lesion extends deeply.

## Aims/Objectives

The aims of this study were to determine the clinical characteristics and operative outcomes of patients who had aortic root abscess. And I introduced a novel surgical option as monobloc aorto-mitral valve replacement for excessive root abscess cases.

## Method

We reviewed 21 patients of active native IE associated with aortic root abscess, and compared with 77 patients of active native IE without aortic root abscess. There was no significant difference between two groups, except logistic Euroscore and peripheral emboli (higher in root abscess).

## Results

Our surgical strategy for active IE is 1) complete debridement of infective tissue, 2) reconstruction with biological material. Six cases of 21 required reconstruction of the aorto-mitral continuity using butterfly shaped bovine pericardial patch in 4, and mono bloc aorta-mitral tissue valves replacement in 2. For the other 15 cases, resected aortic annulus were reconstructed using pericardial patch thereafter reconstruction of aortic leaflets in 6 and tissue valve replacement in 9.

For two cases of excessive root abscess, mitral valve was also infective with vegetation. In these two, after resection of infective tissue including aorto-mitral curtain was completed, Monobloc aorto-mitral biological valve prosthesis was assembled with implanting both aortic and mitral prostheses on a Dacron sheet. The monobloc prosthesis was implanted on the remaining intact aortic and mitral annulus.

In all, septic status was completely disappeared after surgery. In 6 case of annulus reconstruction, the second surgery was required because of residual shunt in 2, PVL in 4. Two cases died from brain infarction in one and congestive heart failure in one.

## Discussion/Conclusion

The Patients with root abscess had higher preoperative comorbidity. Although second cardiac operation was often required, patients with aortic root abscess could be successfully treated with aggressive surgical debridement and LVOT reconstruction. Monobloc combined artificial valve implantation could be one of surgical options for extended aortic annular abscess involving aorto-mitral curtain.

